# Attitudes toward Suicide and the Impact of Client Suicide: A Structural Equation Modeling Approach

**DOI:** 10.3390/ijerph19095481

**Published:** 2022-04-30

**Authors:** Irene Pisnoli, Ruth Van der Hallen

**Affiliations:** Clinical Psychology, Department of Psychology, Education & Child Studies, Erasmus University Rotterdam, 3062 PA Rotterdam, The Netherlands

**Keywords:** practitioner, clinician, survivor, patient, PTSS

## Abstract

Previous research has revealed that mental health professionals (MHPs) often experience significant short- and long-term impacts in the aftermath of client suicide. Individual differences are significant, yet what factors explain these differences remain unclear. The current study aimed to investigate to what extent MHPs’ attitudes toward (client) suicide could predict the short- and long-term impacts of client suicide. A total of 213 MHPs, aged between 18 and 75, reported on a client suicide and their attitudes toward (client) suicide using self-report questionnaires. The results indicate that MHPs who believe it is one’s “rightful choice” to die by suicide report less and MHPs who believe “suicide can and should be prevented” report more impact of client suicide. Predictability and preventability of client suicide proved strongly, positively correlated; yet, neither predicted the impact of client suicide. Taken together, these findings highlight the importance of MHPs’ attitudes toward (client) suicide with respect to clients and MHPs (self-)care.

## 1. Introduction

Suicide, defined as an intentional, self-destructive, and self-inflicted act that causes death, is a challenging public health dilemma worldwide [1]. Each year, between 0.5 and 1.2 million people globally die by suicide [2]. For every single loss, it has been estimated that about 135 individuals consider themselves significantly affected [3]. These “suicide survivors”, i.e., people that shared an emotional connection with a person who died by suicide, include family and friends as well as mental health practitioners (MHPs), whose grief is often disregarded [4]. Considering that mental illness has been argued to play an important role in about 60–98% of all suicides [5,6], high levels of exposure to suicide (loss) in practitioners are not surprising [7]. In fact, practitioners are at the forefront of supporting individuals at risk of suicide, with 30% to 80% of MHPs in Belgium, the U.S., Ireland, and Australia, as well as approximately 98% of MHPs in Slovenia having lost a client to suicide, commonly referred to as “client suicide” [8,9,10].

The impact of client suicide on MHPs has been a topic of investigation since the early 1980s. Research suggests short-term consequences of client suicide may include emotions of shock, disbelief, confusion, and denial, as well as feelings of distress, depression, and anger at the client/society, guilt, shame, a profound sense of responsibility, failure, and feelings of incompetence [11,12]. Post-traumatic distress symptoms, such as intrusive thoughts, avoidant behavior toward potential suicidal clients, sleep disturbances, irritability, difficulty managing life events, and emotional burnout, have been suggested to affect about 50% of MHPs following client suicide [13,14]. Long-term consequences of client suicide may involve feelings of self-doubt and inadequacy, sensitivity to signs of suicidal risk, vigilance and caution when dealing with at-risk patients, concern over one’s competence to treat patients, as well as feelings of anxiety, depression, or helplessness when doing so [14,15,16]. Individual differences regarding the impact of client suicide on MHPs have been associated with differences in gender, age, previous exposure to suicide, or coping strategies [10,14,17,18]. Interestingly, however, it remains unclear to what extent MHPs’ attitudes toward (client) suicide are associated with the impact of client suicide.

Attitudes toward suicide are defined as multidimensional evaluations of the most critical aspects of suicidal behavior as manifested in emotional, instrumental, and cognitive components and can vary widely between individuals (for a review, see [19]). For instance, suicide attempters and suicide contemplators have been found to be more accepting of suicide than non-attempters or people without a history of suicidal ideation [20,21]. Furthermore, people with more permissive attitudes toward suicide have been associated with greater rates of suicide ideation [22]. More recently, Pitman and colleagues [23] conducted a qualitative study of attitudes toward suicide in 429 young bereaved adults and found that exposure to the suicide of a close friend or relative can influence attitudes to suicide in ways that would influence one’s own risk of suicide later in life. The attitudes of medical staff toward suicide have been known to affect the care they provide suicidal patients [24]. Moreover, Samuelsson and colleagues [25] found nurses’ willingness to treat and their ability to empathize with suicidal patients depended on their attitudes toward suicide.

MHPs’ attitudes toward suicide, such as the attitudes of psychologists, psychotherapists, psychiatrists, or social workers, have not been researched extensively. Werth and Liddle [26] investigated attitudes toward suicide in 186 psychotherapists and found significant individual differences in accepting suicidal ideation as well as actions taken to prevent a suicide depending on why a person had decided to die by suicide. Overall, psychotherapists with more experience were found to be more accepting of suicide and to take less action to prevent suicide than less experienced psychotherapists. Swain and Domino [27] investigated attitudes toward suicide in 1441 mental health professionals. Overall, clergy and general physicians were found to be less accepting of suicide, especially when compared to social workers, who were found to be the most accepting of suicide. Moreover, professionals with personal experience or acquaintance with suicide were found more likely to accept the notion of suicide and better able to recognize signs of suicidal ideation [27,28,29]. That being said, one can wonder if one’s attitude toward suicide might not just dictate how we deal with, treat, assess, or intervene in cases of suicide, but also how we cope or deal when faced with a client’s suicide.

Therefore, the current study aims to investigate to what extent MHPs’ attitudes toward suicide are associated with the impact of client suicide. In other words, to what extent are MHPs’ attitudes toward (client) suicide informative regarding the short- and long-term emotional and professional impacts of client suicide? The results of this study may help broaden our understanding of the effects of a client’s suicide as experienced by MHPs, providing insights relevant for training purposes or to reduce professional stigmatization following a client loss.

## 2. Method

### 2.1. Participants

This study included data from 213 participants (25% male, 72% female, and 1% non-binary) aged between 18 and 75 years. All participants had experienced at least one client suicide. The majority of the sample originated from Belgium (47%), Germany (18%), or The Netherlands (15%). A total of 46% of the participants were psychologists, 14% were psychiatric nurses, 13% were psychiatrists, 10% were counselors, and 9% were social workers.

### 2.2. Procedure

The present study is part of a larger research project looking into the impact of client suicide (for more details, see [17]). All study protocols were in accordance with the Declaration of Helsinki and approved by the Ethics Committee of the Erasmus University Rotterdam, The Netherlands (19-007.R1). The study was conducted via an online survey and was aimed at MHPs who had experienced client suicide. Recruitment was set up via social media, professional newsletters, and email. Individual informed consent was obtained from all participants involved in the study prior to participation. Data were collected using a self-administered, online survey available in English, Dutch, and German. All participants completed two questionnaires regarding their attitudes toward (client) suicide as well as three questionnaires related to the impact of client suicide (for more detail, see below). Survey completion took approximately 15–20 min.

### 2.3. Materials

The ATTS-18 [30] is an abbreviated version of the Questionnaire on Attitudes Toward Suicide [31], a self-report questionnaire developed to assess one’s attitudes toward suicide. Example items include: “*people do have the right to take their own lives*” and “*if someone wants to commit suicide it is their business and we should not interfere*”. Each item is rated on a 5-point Likert scale, ranging from 1 “*strongly disagree*” to 5 “*strongly agree*”. Following EFA/CFA of the ATTS-18, construct reliability was evaluated for our subscales (see Results).

To assess participants’ attitudes regarding the predictability or preventability of client suicide, inspired by Alexander and colleagues [15], the following two sets of questions were included. In general, “how predictable is client suicide?”, and “how preventable is client suicide?” (labeled as Pred1 and Prev1). In reference to one particular client’s suicide, “how predictable was that client’s suicide?” and “how preventable was that client’s suicide?” (labeled as Pred2 and Prev2). Either item is rated on a 5-point Likert scale, ranging from 1 “*very unpredictable*” to 5 “*very predictable”* or 1 “*very unpreventable*” to 5 “*very preventable*”.

The IES-22-R is a revised version of the original IES [32,33], a self-report questionnaire that aims to measure the subjective stress of a particular (traumatic) event in the seven days following. It includes 22 items divided over 3 subscales: (1) intrusion, (2) hyperarousal, and (3) avoidance. Each item is rated on a 5-point Likert scale, ranging from 0 “*not at all*” to 4 “*extremely*”. All 22 items are included in a total sum score. Cronbach’s alpha as calculated for the current sample was α = 0.95, suggesting excellent scale reliability.

The Long-Term Emotional Impact Scale (LTEIS; [34,35]) is a self-report questionnaire that aims to measure the long-term emotional impact of MHPs who have experienced client suicide. It consists of 10 items focusing on negative emotions that can occur following client suicide. Examples include aspects of therapeutic competence, such as a diminished sense of personal effectiveness, increased anxiety when evaluating suicidal clients, or the evaluation of a greater number of clients as being at risk of suicide. Each item is rated on a 5-point Likert scale, ranging from 1 “*disagree*” to 5 “*agree*”. All 10 items are included in a total average score. Cronbach’s alpha as calculated for the current sample was α = 0.87, suggesting good scale reliability.

The Professional Practice Impact Scale (PPIS; [34], inspired by [36]) is a self-report questionnaire that aims to measure long-term changes in professional practices in MHPs who have experienced client suicide. It consists of nine items that focus on the changes in professional practice that often follow client suicide, such as the refusal to work with suicidal clients, a greater inclination to consult colleagues, or the consideration of leaving the profession because of client suicide. Each item is rated on a 5-point Likert scale, ranging from 1 “*disagree*” to 5 “*agree*”. All nine items are included in a total average score. Cronbach’s alpha as calculated for the current sample was α = 0.77, suggesting good scale reliability.

### 2.4. Data Analysis

Statistical analyses included exploratory factor analysis (EFA) and structural equation modeling (SEM) using IBM SPSS Statistics 27.0 and AMOS 28.0 for Windows. EFA analyses were performed in SPSS using principal axis factoring as an extraction method, with oblimin rotation and Kaiser normalization. To determine the best factor structure, the eigenvalues (>1), factor loadings (≥0.4), scree plot, and conceptual coherence of the individual factors were taken into account [37]. SEM analyses were performed in AMOS using the maximum likelihood estimation method. Global model fit was evaluated using the comparative fit index (CFI; CFI ≥ 0.90) and root mean square error of approximation (RMSEA; 0.05 ≥ RMSEA ≤ 0.08) [38,39].

## 3. Results

To investigate the extent to which one’s attitudes toward suicide, as measured by the ATTS-18, are associated with the short- (IES-R) and long-term (LTEIS and PPIS) impacts of client suicide, EFA and path analysis in SEM were conducted. First, the ATTS-18 was explored using EFA. Factor loadings for the 18 items of the ATTS-18 and their item descriptions are presented in Table 1. EFA analysis identified a two-factor structure accounting for 45.12% of the total variance. Factor 1 was defined as *Rightful Choice* (nine items) and accounted for 32.28% of the variance with an eigenvalue of 5.81. Factor 2 was defined as *Preventability* (six items) and accounted for 12.84% of the variance with an eigenvalue of 2.31. Item 1, Item 8, and Item 17 were removed from the model as they did not load sufficiently on either factor (<0.40). Next, this two-factor structure was confirmed using SEM. To obtain a good model fit, guided by the modification indices and the correlation matrix, Item 12 and Item 13 were removed from the model, and an error correlation between Items 9 and 15 was included. Acceptable model fit, with CFI = 0.91 and RMSEA = 0.08, 90% CI (0.068–0.092), was achieved for a model with two first-order latent variables (i.e., *Rightful Choice*, seven items, and *Preventability*, six items).

**Figure 1:**
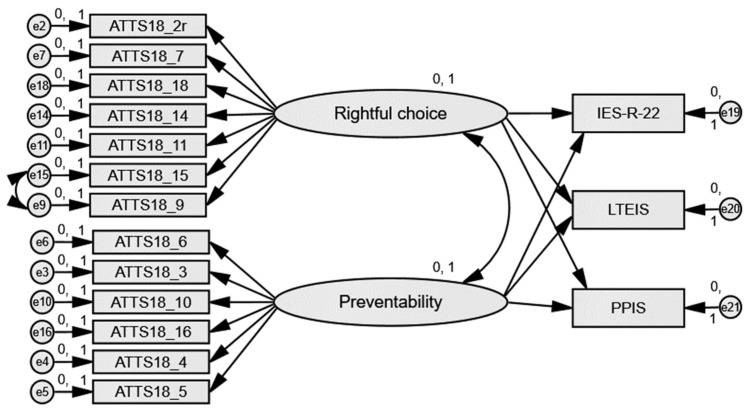
Path analysis for the short-term (IES-R), long-term emotional (LTEIS), and long-term professional (PPIS) impacts of client suicide, as predicted by the ATTS-18.

Next, a path analysis in SEM was constructed to evaluate the extent to which the two-factor structure of the ATTS-18 was able to predict the short- and long-term impacts of client suicide (see Figure 1 and Table 2). The two-factor model explained 14% of short-term, 7% of long-term emotional, and 12% of long-term professional impact variance. Rightful Choice and Preventability were both significantly related to all three impact variables (*p* < 0.05). Specifically, Rightful Choice had a negative significant relationship with short-term (*β* = −0.31, *p* < 0.001), long-term emotional (*β* = −0.22, *p* = 0.017), and long-term professional (*β* = −0.19, *p* = 0.037), whereas Preventability had a positive significant relationship with short-term (*β* = 0.42, *p* < 0.001), long-term emotional (*β* = 0.29, *p* = 0.002), and long-term professional (*β* = 0.40, *p* < 0.001). In other words, to hold the view that it is one’s rightful choice to complete suicide is associated with less impact of client suicide, whereas to hold the view that suicide is (and should be) prevented is associated with more impact of client suicide.

To investigate the extent to which MHPs’ attitudes toward client suicide are associated with the short- (IES-R) and long-term (LTEIS and PPIS) impacts of client suicide, CFA and path analysis were conducted. First, the two-item, two first-order latent variable factor structure (i.e., Predictability and Preventability) was confirmed using SEM. Since both latent variables contain only two indicators, factor loadings were constrained to be equal prior to analysis [40]. Good model fit, with CFI = 0.97 and RMSEA = 0.05, was achieved.

**Figure 2:**
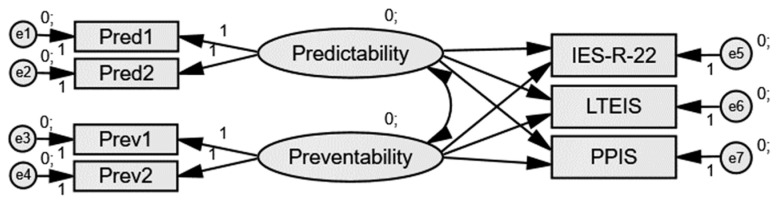
Path analysis for the short-term (IES-R), long-term emotional (LTEIS), and long-term professional (PPIS) impact of client suicide as predicted by Predictability vs. Preventability.

Second, a path analysis in SEM was constructed to evaluate the extent to which the two-factor structure was able to predict short- and long-term impacts of client suicide (see Figure 2 and Table 3). The model explained 44% of short-term, 65% of long-term emotional, and 63% of long-term professional impact variance. Predictability and Preventability were highly positively correlated (*r* = 0.97, *p* <0.001), yet neither proved significantly related to any of the three impact variables (*p* > 0.05). Predictability was not significantly related to short-term (*β* = −2.43, *p* = 0.225), long-term emotional (*β* =−3.04, *p* = 0.213), or long-term professional impact (*β* = −2.94, *p* = 0.220). Similarly, Preventability was not significantly related to short-term (*β* = 2.54, *p* = 0.190), long-term emotional (*β* = 3.08, *p* = 0.191), and long-term professional impact (*β* = 3.04, *p* = 0.189). In other words, MHPs’ attitudes toward (a particular) client suicide were not associated with the impact of said client suicide.

## 4. Discussion

Individual differences regarding the impact of client suicide on MHPs have long been a topic of investigation, for instance, with regard to the difference in gender, age, previous exposure to suicide, or coping strategies (e.g., [10,14]). Interestingly, the extent to which one’s attitudes toward (client) suicide might be associated with the impact of client suicide on MHPs had not received a lot of attention. Therefore, the current study aimed to investigate to what extent MHPs’ attitudes toward (client) suicide are informative regarding the short- and long-term impacts of client suicide. Looking at attitudes toward suicide, our model explained 14% of short-term, 7% of long-term, and 12% of the long-term professional impact variance. Rightful Choice was associated with less short- and long-term impact, whereas Preventability was associated with more short- and long-term impact. Looking at attitudes toward client suicide, our model explained 44% of the short-term, 65% of the long-term emotional, and 63% of the long-term professional impact variance. Yet, neither Predictability nor Preventability predicted impact. Implications for both research and clinical practice are discussed.

Regarding attitudes toward suicide, our results indicate that generally, one’s attitudes toward suicide can indeed play an important role in understanding individual differences in impact following client suicide. Rightful Choice was negatively associated with short- and long-term impact, whereas Preventability was positively associated with short- and long-term impact. In other words, participants who hold the belief that “one has the right to take their own life”, “would consider the possibility if (…)”, or “can understand that people complete suicide” reported less impact of client suicide, whereas participants who hold the belief that “suicide can be prevented”, “it is our human duty to prevent (…)”, or “(suicide) is among the worst things to do (…)” reported more impact of client suicide. Whilst novel, overall, these findings seem in line with previous research that suggests that our attitudes toward suicide impact how we perceive suicide-related behavior or intentions. As aforementioned, individuals with a history of suicidal ideation or non-fatal suicide attempts have been found to be more accepting [20,21] and individuals with more permissive attitudes toward suicide have been associated with greater rates of suicide ideation [22]. Moreover, exposure to the suicide of a close friend or relative has been found to influence one’s attitudes toward suicide [23], and attitudes of medical staff toward suicide have been known to affect the (willingness to and) care provided to suicidal patients [24,25]. Last but not least, the belief that “any and every suicide is preventable” has been associated with increased distress in MHPs, as MHPs and their organizations involved in a client suicide may therefore be more inclined to look for a scapegoat or direct blame to one person [41].

Regarding attitudes toward client suicide, our results reveal that the more one considers client suicide predictable, the more preventable one considers it to be, and the other way around; yet, neither stance was significantly associated with the impact of client suicide. Previous research considering attitudes toward client suicide is limited and mostly descriptive. Rothes and colleagues [10] investigated the impact of client suicide in 107 psychiatrists and revealed that 57% of psychiatrists considered their most distressing case of client suicide to be little or not at all preventable and 39% of them thought the event was little or not at all predictable. As such, the predictability and preventability expectations of client suicide seemed to be associated with subsequent distress. Moreover, while Alexander and colleagues [15] reported that publicity in the media and the prospect of litigation exacerbated or modulated the impact of client suicide, attitudes toward client suicide did not. Interestingly, however, the authors did note the importance of said attitude, concluding that “*psychiatrists have to strike a difficult balance in their attitudes to suicide. If they regard suicide as fundamentally unavoidable (…) such a belief may foster therapeutic nihilism, (…) if suicide is perceived to be largely preventable and predictable, this may foster a culture of blame.”* [15] (p. 1573). Last but not least, previous research suggests that if one can recognize that it is the client, not the MHP, who is ultimately responsible for a client’s suicide, this ameliorates the impact of client suicide on the mental health professional [35,42].

Important implications for clinical practice (and supporting research) follow from these results. The current results suggest our attitudes toward (client) suicide do not only influence how we think about client suicide, suicidality, or the care we provide, but also to what extent we are affected or impacted by a client’s suicide. Currently, however, (post)graduate training programs for MHPs pay little to no attention to one’s attitudes toward suicide [43]. If within these programs suicide is discussed, the emphasis is on understanding suicide, its prevention, and care, and rarely are MHPs asked to reflect on their own ideas or attitudes toward suicide, nor are they stimulated to consider the relevance of such beliefs [44]. Helping MHPs become more aware of the (importance) of their attitudes toward suicide may prove valuable with regard to prevention, (self-)care, and MHPs’ understanding of what they may (not) experience in the aftermath of client suicide. Individual differences in how MHPs respond to client suicide are significant and often puzzling to all parties involved. Increased awareness of what factors underlie these individual differences may prevent stigma, maladaptive emotional responses, or feelings of loneliness and isolation. This proposition is in line with previous work by Linke and colleagues [45], who have suggested that MHPs’ professional training should foster the idea that suicides are not always preventable. Similarly, Sanders and colleagues [46] have argued in favor of exploring feelings of powerlessness that may arise following a client’s suicide and of the notion that not all suicides can be prevented. While the goal would not be (and should not be) to change MHPs’ attitudes toward suicide, conversations about the relevance of one’s attitudes toward suicide among fellow students or colleagues may prove highly valuable for both patient care as well as self-care regarding suicidality. Moreover, suicide-related training programs should educate MHPs on the likelihood or probabilities of (client) suicide, that suicide is not always preventable, and what the personal and professional impact of a client’s suicide might involve. The vast majority of MHPs do not typically receive suicide-related training in assessment, treatment, or risk management, while such programs have been found to positively impact professional practices, clinic policy, and clinicians’ confidence and beliefs [47,48].

### Limitations and Recommendations

The design of the current study was cross-sectional and employed a convenience sample. Moreover, retrospective self-report data were collected and analyzed. An implication of this would be that the events, thoughts, feelings, and behaviors reported by the participants could have taken place at any time, even many years previously, and may be subject to self-report bias. Although self-report questionnaires and convenience samples are frequently used in trauma research (e.g., [49,50,51,52,53]), the generalizability of the results may be limited as a result. Moreover, MHPs’ attitudes toward suicide were collected when at least one client suicide had already taken place (see inclusion criteria for the current study), rather than before (and after) such event had taken place. The number of client suicides experienced, or the time passed since the event, were not recorded. Previous research, however, suggests that experiencing a client’s suicide can alter a clinician’s attitudes toward suicide [26,28,29,54]. As such, the current results pertain only to the relationship between MHPs’ attitudes toward (client) suicide and the impact of said client suicide as measured after the fact and may not entirely generalize to attitudes toward (client) suicide measured when no such client suicide has taken place (yet). Finally, the ATTS-18 included in the current study was subject to an EFA, resulting in two distinct factors not identified by others previously, potentially limiting the generalizability of the results. Future research is advised to employ a longitudinal design and/or employ a more qualitative approach, assessing attitudes toward (client) suicide in MHPs at the start of their career, allowing for the (re)evaluation of said attitudes over time and, if the situation occurs, following one or more client suicides. That way, research could also further investigate to what extent the number of client suicides one has experienced impacts both MHPs’ attitudes toward suicide as well as the impact of client suicide [34]. Moreover, qualitative approaches would allow for a deeper understanding of the experience the MHP has gone through and of the correlated emotions in the MHPs’ own words.

## 5. Conclusions

To conclude, the current study conducted an in-depth investigation of MHPs’ attitudes toward (client suicide) in light of the short- and long-term impacts of client suicide. Overall, the results indicate that MHPs who believe it is one’s “rightful choice” to complete suicide tend to report less impact of client suicide, whereas MHPs who believe “suicide in general can and should be prevented at all times” tend to report more impact of client suicide. Moreover, the more MHPs consider client suicide as predictable, the more preventable MHPs consider it to be, and the other way around. As such, the current findings highlight the importance of suicide-related training programs and the extent to which such training programs should discuss MHPs’ attitudes toward (client) suicide to improve client care as well as MHPs’ self-care and mental well-being.

## Figures and Tables

**Table 1:** Factor loadings for the 18 items of the ATTS-18.

		Component	
		1	2
14.	People do have the right to take their own lives.	0.760	
02.	Suicide can never be justified. (R)	0.741	
09.	I would consider the possibility of taking my life if suffering from a severe, incurable disease.	0.720	
11.	A person suffering from disease expressing wishes to die should get help to do so.	0.693	
13.	I can understand that people suffering from a severe, incurable disease commit suicide.	0.688	
07.	There may be situations where the only reasonable resolution is suicide.	0.671	
15.	I would like to get help to commit suicide if I were to suffer from a severe, incurable disease.	0.636	
12.	I am prepared to help a person in a suicidal crisis by making contact.	0.601	
08.	Although you would prefer to die in a different way, encountering painful life circumstances could make you consider suicide.	0.565	
18.	Suicides among young people are particularly puzzling since they have everything to live for.		
17.	Suicide should not always be prevented.		
10.	If someone wants to commit suicide it is their business and we should not interfere. (R)		0.774
04.	Once a person has made up their mind about suicide no one can stop them. (R)		0.670
05.	It is a human duty to try to stop someone from committing suicide.		0.665
16.	Suicide can be prevented.		0.651
03.	Committing suicide is among the worst things to do to one’s relatives.		0.515
06.	Loneliness could for me be a reason to take my life. (R)		0.471
01.	It is always possible to help a person with suicidal thoughts.		

Extraction method: principal axis factoring. Rotation method: oblimin with Kaiser normalization.

**Table 2:** Regression weights for the ATTS-18 predicting short- and long-term outcomes of client suicide.

		Unstandardized Estimate	Standard Error	Standardized Estimate	*p*-Value
Rightful Choice	IES-R	−6.020	1.734	−0.314	<.001
Rightful Choice	LTEIS	−0.199	0.083	−0.217	.017
Rightful Choice	PPIS	−0.135	0.065	−0.187	.037
Preventability	IES-R	8.019	1.778	0.418	<.001
Preventability	LTEIS	0.266	0.085	0.291	.002
Preventability	PPIS	0.290	0.067	0.401	<.001

Note. IES-R: Impact of Event Scale—Revised; LTEIS: Long-Term Emotional Impact Scale; PPIS: Professional Practice Impact Scale.

**Table 3:** Regression weights for Predictability and Preventability predicting short- and long-term outcomes of client suicide.

		Unstandardized Estimate	Standard Error	Standardized Estimate	*p*-Value
Predictability	IES-R	−88.962	73.257	−2.432	.225
Predictability	LTEIS	−5.288	4.242	−3.038	.213
Predictability	PPIS	−4.050	3.301	−2.940	.220
Preventability	IES-R	177.257	135.131	2.540	.190
Preventability	LTEIS	10.217	7.818	3.076	.191
Preventability	PPIS	7.992	6.089	3.040	.189

Note. IES-R: Impact of Event Scale—Revised; LTEIS: Long-Term Emotional Impact Scale; PPIS: Professional Practice Impact Scale.

## Data Availability

The data that support the findings of this study are available on request from the corresponding author, Ruth Van der Hallen.
